# On the Relationship Between English as a Foreign Language Learners’ Positive Affectivity, Academic Disengagement, and Communication Apprehension

**DOI:** 10.3389/fpsyg.2021.828873

**Published:** 2022-02-15

**Authors:** Yuxia Ma

**Affiliations:** English Department, School of Foreign Languages and Cultures, Chengdu University, Chengdu, China

**Keywords:** positive psychology, academic disengagement, communication apprehension, positive affectivity, positive classroom context

## Abstract

This review tends to investigate the related studies on the relationships among positive affectivity as a type of positive psychology construct and academic disengagement and communication apprehension (CA) as two types of negative emotions. The negative correlations among CA, disengagement, and positive affectivity like enjoyment have been verified in the review of the literature. Moreover, little research has been done on the relationship between academic disengagement and CA. The studies showed that some factors such as encouraging teaching methodologies, positive classroom context, exciting and challenging classroom tasks can act as mediators in the relationship between positive affectivity and CA. In the end, the pedagogical implications are explicated to foster the language learning quality and to develop a language educational system. Suggestions for further research are provided to develop the existent literature on the relationship between English as a foreign language (EFL) learners’ academic disengagement, CA, and their positive affectivity.

## Introduction

Positive psychology has drawn the attention of many investigators in foreign language learning since it tries to enhance learning outcomes and foster learning contexts (Han, 2021; Wang et al., 2021). The development of positive psychology owes to Seligman’s (2011) theorization of “well-being.” He developed five-dimensional PERMA including “positive emotion (P), engagement (E), relationships (R), meaning (M), and accomplishment (A)” (p. 12). The enhancement of all five dimensions is the objective of positive psychologists (Wang et al., 2021). On the other hand, the processes of learning a foreign language can be a demanding experience for some learners, and learners’ individual differences have been studied in terms of socio-demographic and affective factors such as self-efficacy, apprehension, personality, motivation, and self-efficacy (Kim et al., 2015). More studies need to be done on this issue. This review aims to scrutinize the relationships among apprehension, positive emotions, and engagement in English as a foreign language (EFL) contexts.

## Literature Review

### Apprehension

Gardner and MacIntyre (1993) pointed out that “Anxiety is fear or apprehension occurring when a learner is expected to perform in the second or foreign language” (p. 59). They linked it to the stimulation of the autonomic nervous system. In a foreign language learning context, Horwitz et al. (1986) coined the term “foreign language anxiety” (FLA) and defined it as “a distinct complex of self-perceptions, beliefs, feelings, and behavior related to classroom language learning arising from the uniqueness of the language-learning process” (p. 28). FLA has been widely considered as a leading factor in academic achievement and language proficiency (MacIntyre, 2017). Studies have testified that higher levels of anxiety are negatively correlated with foreign language proficiency, and with positive orientation and peer emotional support (Zheng and Cheng, 2018). Horwitz (2017) also found out that FLA has a negative significant correlation with some affective factors such as learners’ motivation, willingness to communicate, and their self-esteem. Horwitz et al. (1986) categorized FLA construct into Test Anxiety, Fear of Negative Evaluation, and communication apprehension (CA). CA, defined as the avoidance of communications with others (Bourhis and Allen, 1992), has been still widely studied in recent years in communication research (Sham and Azmi, 2018). CA, as a focal point of this review, has a significant correlation with language learners’ linguistic background and their proficiency levels (Molnar and Crnjak, 2018). Spetz (2018) investigated Swedish foreign language learners’ CA and he attributed it to the level of proficiency in language learners as beginners have a higher level of CA.

### Academic Disengagement

Learners’ academic disengagement is one of the most important problems in academic contexts (Lin, 2017). Skinner et al. (2009) defined disengagement as “the absence of engagement including the absence of effort or persistence” (p. 495). Academic engagement is described as “learners’ psychological effort and investment toward learning, understanding, or mastering the skills, crafts, or knowledge that the coursework is intended to promote” (Lamborn et al., 1992, p. 13). Learners’ academic engagement includes individual and contextual features that interact with each other to demonstrate their positive attitudes toward learning (Zhao et al., 2021). Eccles (2016) argued that learner academic engagement has a positive and significant relationship with academic achievement and resilience. Dörnyei and Ushioda (2011) considered learner motivation a precondition for learner engagement and academic achievement. Gurian et al. (2011), in their study, found out that gender is correlated with differences in learners’ academic engagement as males’ and females’ brain structures differ. Morisse (2015) revealed that females are more academically engaged than males. Rabourn et al. (2015) highlighted the influence of age on learner engagement, and they asserted that adult learners (over 21) have higher academic engagement levels than younger ones. Hashim et al. (2014) also demonstrated that the interaction and rapport between teacher and learner influence learners’ engagement. Guilloteaux (2016) asserted that learners’ engagement is affected by their background level, task type and difficulty, instructors’ methodology and their motivation, and their teaching style. Hung (2015) argued that memorization and rote learning, as two outdated teaching approaches, significantly predict learners’ disengagement. He suggested flipped instruction for solving disengagement. Gunuc (2014) also maintained that learner disengagement leads to indifference and frustration, which restrain academic performance.

### Positive Affectivity

According to Agudo (2018), emotions are “subjective, evaluative judgments through which we attempt to interpret the situations we find ourselves in” (p. 386). Traditionally, most investigations have highlighted negative emotions, particularly anxiety. However, with the advent of positive psychology in foreign language studies, most investigators tend to study the facilitative influence of positive affectivity or emotion in foreign language teaching (White, 2018). Pekrun (2014) found out that positive affectivity is significantly related to the learning tasks, intrinsic motivation, the use of language learning strategies, and achievements. Schunk and Greene’s (2018) study revealed that the interaction between positive affectivity and self-regulation may result in achievement in language learning, their well-being, and their happiness. Pekrun (2014) has introduced four types of positive emotions that arise during learning activity or outcome: enjoyment, hope, pride, and relief. Goetz et al. (2007) studied the relationship between positive affectivity factors including enjoyment and pride, and negative ones including anger and boredom in various domains, such as English, German, mathematics, and physics classrooms. Their study revealed the context-based differences in the relationships between positive and negative affectivity. They also found stronger relationships between positive and negative emotions in similar subject domains.

### The Relationship Between Positive Affectivity and Apprehension

The relationship between positive affectivity and CA in international educational contexts has been widely studied (Dewaele and MacIntyre, 2014). Enjoyment as one type of positive affectivity has drawn the attention of many investigators since the publication of Dewaele and MacIntyre’s (2014) study. Other types of positive affectivity such as pride and hope are not widely studied which can pave the way for future studies. Boudreau et al. (2018) reported enjoyment as one of the most regular phenomena experienced in individuals’ life. They defined enjoyment in a foreign language context as a “complex and stable emotion that is completely separate from the more superficial experience of pleasure” (p. 153). Dewaele (2017) mentioned that enjoyment and anxiety have been theorized as two distinct but related aspects in foreign language contexts. They are negatively correlated with each other. The reason for the negative correlation is teachers’ approach in increasing foreign language enjoyment and decreasing foreign language apprehension. They can provide positive emotional EFL contexts to increase foreign language enjoyment and decrease apprehension levels.

Dewaele and MacIntyre (2014) found a significant negative correlation between enjoyment and CA. Their study also revealed that enjoyment and apprehension or anxiety are not two conflicting constructs. However, a learner may not enjoy in the foreign language context but still have no level of CA (Dewaele and MacIntyre, 2014). Moreover, Dewaele et al. (2018) asserted that learners with significantly high levels of enjoyment and low levels of CA in foreign language contexts had higher levels of language proficiency. Li et al. (2020) also pinpointed the significance of enjoyment and apprehension as the predictors of EFL proficiency at different achievement levels. They attributed this result to the reason that learners with a low level of foreign language apprehension and a high level of enjoyment tend to be more confident and more optimistic which results in higher performance in foreign language proficiency.

Resnik and Dewaele (2020) also studied enjoyment and CA among the first language (German participants) and foreign language learners (English participants). Their study revealed that, in a foreign language context, higher levels of apprehension and enjoyment were observed. Furthermore, there was a negative correlation between enjoyment and apprehension in first and foreign language contexts. They attributed their results to the use of educational approaches in foreign language classrooms may lead to higher levels of apprehension and enjoyment. Pavelescu and Petrić (2018) provided some factors such as peer interaction, supportive and encouraging teaching, positive classroom context, exciting and challenging classroom tasks which play as mediator variables between apprehension and enjoyment in EFL contexts. These variables should be scrutinized in more detail to clarify the relationship between enjoyment and apprehension in foreign language contexts.

Pride, as another aspect of positive affectivity, has received little attention (Artino and Jones, 2012). Heckel and Ringeisen (2019) investigated pride and apprehension and learners’ self-efficacy, interest, and satisfaction in online learning contexts. Their study showed a negative significant relationship between self-efficacy and apprehension and a positive significant relationship between pride and self-efficacy, interest and satisfaction. They found a negative relationship between pride and anxiety and they attributed this result to the fact that pride depends on dispositional control along with contextual value appraisals, while apprehension is mainly related to dispositional control appraisals.

### The Relationship Between Engagement, Disengagement, and Positive Affectivity

Most studies have been done on the influence of learners’ and instructors’ individual differences and their relations with learners’ engagement in foreign language contexts and their linguistic proficiency (Xie and Derakhshan, 2021). A significant correlation between learner engagement and teacher-student rapport and non-verbal immediacy, as two elements of positive psychology, has been identified (Derakhshan, 2021; Greenier et al., 2021). However, the related studies ratified the implication of promoting engagement in foreign language learning contexts since it triggers two components of positive psychology like learners’ motivation and enjoyment and results in learners’ academic achievement (Dewaele and Li, 2020; Zeng, 2021). Mercer and Dörnyei (2020) argued that foreign language learners’ enjoyment positively correlates with their engagement in language learning contexts. They also maintained that learners’ enjoyment of foreign languages can support their academic engagement. Learners are enthusiastically, interactively, and cognitively involved in language learning than genetically. They ascribed their results to the custom of venerating the teacher and valuing his teaching in the educational context.

Jin and Zhang (2019) asserted that language learning enjoyment can lead to engagement in learning contexts and it can improve social-behavioral learning engagement. They emphasized that enjoyment may lead to constant willpower along with engagement in instructive contexts. Liu (2021) investigated the way of developing learners’ positive affectivity by increasing their engagement. He indicated that learner enjoyment, arising from peer relations, teacher-instructor rapport and the difficulty of the tasks is a significant element to support learner engagement. Philp and Duchesne (2016) regarded engagement as a determining factor for motivation and they argued that engagement is an apparent indicator of cognitive and emotional behavior in the form of enjoyment and it influence effort and strategies for learning.

Regarding disengagement, Chipchase et al. (2017) identified attendance, preparation for class, time and effort spent studying, collaborative study, assessment, academic performance, and enjoyment as the indicators of academic disengagement. Most of these indices are behavior and cognitive reasons for disengagement and only lack of enjoyment is the psychological reason for disengagement. These variables can offer a basis for the preparation of a screening tool to gauge learner disengagement. These variables should be considered in more detail in future studies and the reasons for academic disengagement should be meticulously studied. Overall, positive affectivity is related to intensified engagement, whereas negative affectivity is associated with academic disengagement. Particularly, enjoyment promotes engagement (Ainley and Ainley, 2011).

### The Relationship Between Engagement, Disengagement, and Apprehension

According to Dörnyei and Ushioda (2011), learning a foreign language includes interaction between motivation, cognition, and affection. Since individual differences are characterized by “highly interrelated, multifaceted, complicated, and dynamic” (Butler, 2019, p. 4), it can be mentioned that CA or anxiety and academic engagement have direct and indirect relationships with each other (Zhang et al., 2020). Few studies have been done on the effect of engagement on anxiety. Kashdan and Fincham (2004), in their study, found out that anxiety and disengagement happen when academic problems are observed. Zhang et al. (2020) in their study, found out that L2 motivators outperform L2 demotivators in predicting learners’ engagement in EFL contexts and their CA. Generally, they pointed out that the consideration of methodologies used in classroom and testing approaches can support L2 motivation and learners’ academic engagement. They maintained that engagement acts as a mediator role in L2 de/motivation, whereas, CA may hold back learners’ academic achievement and improve their intention to engage in the EFL classrooms. Li (2021) also argued apprehension can bring about that permanent harm in learners’ performance since anxious learners may not have many chances to express themselves and to engage themselves in language learning contexts.

## Discussion

This review tried to investigate the related literature reviews on apprehension and disengagement as negative emotions. However, positive affectivity, especially enjoyment, has been meticulously studied. This review scrutinized the studies related to the negative relationship between enjoyment and apprehension. However, some factors such as learners’ confidence, negative and positive attitudes and teachers’ educational methodologies can as mediator variables between learners’ classroom enjoyment and their apprehension. This review also highlighted the studies which prove the significant relationship between enjoyment and engagement in foreign language contexts. We can attribute this positive correlation to peer relations, teacher-instructor rapport, and challenging tasks as they increase learners’ feeling of enjoyment, competitiveness, and academic engagement. Few studies also considered a negative relationship between apprehension and engagement. In other words, the more anxious a learner feels, the less engaged they are to probably join in class activities. More studies are required to consider this correlation in more detail both among learners and teachers.

### Implications and Suggestions

This review investigated the correlations between learners’ communicative apprehension, engagement, and positive affectivity. Considering the related studies on the relationship among affective factors, it can be mentioned that learners should be assisted to control, adjust, and regulate their feelings in language learning contexts. Lack of regulating or controlling affections may diminish the enjoyment of learners which may trigger teacher educators to consider this issue in practical aspects (Schutz and Zembylas, 2009). The review of literature can motivate instructors, school principals, and policymakers to ponder into EFL learners’ behaviors, characteristics, and their positive effects. The literature implied that positive affectivity such as enjoyment and pride and negative emotions such as academic disengagement and apprehension or anxiety occur in L2 classes.

This review implies that instructors can modify learners’ academic enjoyment, engagement and lessen their apprehension by providing a peaceful context for language learning. Therefore, L2 instructors require courses to enhance learners’ attitudes and motivation for increasing their positive affectivity such as foreign language enjoyment, engagement, pride, etc., and to alleviate negative feelings such as CA, disengagement, etc. in their classes. Furthermore, the knowledge of EFL learners’ characters may inspire instructors to be more stable and engaging in their behavior in language contexts. They can provide learners with chances for foreign language enjoyment, and they are required to diminish learners’ CA, disengagement, tension and improve resilience despite educational problems in language learning contexts to enhance L2 learning experiences. They can provide foreign language input by boosting student-student interactions. In a foreign language context, learners’ CA is related to their language proficiency level and their linguistic background (Molnar and Crnjak, 2018), therefore homogenizing the classroom can be an appropriate approach to deal with this problem. Furthermore, instructive supervisors who monitor instructors and assess their academic efficiency can exploit the related studies through consideration of the instructors’ interpersonal behaviors and their rapport with the learners. Policymakers can develop engagement programs that help learners decrease their CA and amplify their academic engagement. They can positively support learners and make a context in which learners can take part in positive behaviors.

The schools and institutes’ managers should provide EFL contexts in support of learners’ engagement and enjoyment by offering to authenticate, joyful and updated materials to teachers and learners. They can recommend teachers to use interesting teaching methodology and styles which arouse learners’ excitement and enjoyment to engage in the classroom. Given that the learners’ academic engagement is a primary facet of education, the anticipation of strategies for intensifying learners’ enjoyment and engagement in pre-service and in-service teacher training programs by teacher educators can be valuable. Also, it is recommended that school managers hold instructive workshops bearing in mind learners’ apprehension, their enjoymentt, and academic engagement. Lastly, the importance of academic engagement and enjoyment makes advisors widen agendas to upsurge the influence of these academic engagements and enjoyment on learning achievement. They can identify learners’ sources of academic disengagement.

This review has raised numerous questions for further investigations. Future studies may consist of investigating the influence of other individual variables including learner’s extroversion, introversion, and many others. The causes of positive affectivity and CA should be investigated to reveal the primary causes for classroom emotions. Moreover, reciprocity of the relationship between foreign language affectivity and apprehension should be studied in the future. Other studies can be done to investigate which linguistic tasks can alleviate learners’ apprehension and their academic disengagements. Longitudinal studies are required to shed light on the intrapersonal and interpersonal emotions in language learning. Dörnyei and Ryan (2015) also suggested the requirement of longitudinal and dynamic research approaches that show learners’ changing nature in emotions in language learning contexts. Future studies can investigate the effects of positive psychology constructs on the working memory of EFL learners. Moreover, the effects of positive psychology constructs on the improvement of language skills should be studied in detail. Language learning *via* online language teaching has been disturbed severely during the COVID-19 pandemic in the EFL context (Wang and Derakhshan, 2021). Further studies are needed to determine learners’ academic engagement, CA, and their positive affectivity in traditional and digital contexts to illuminate how these contexts may affect learners’ emotions (Wang and Guan, 2020). Furthermore, further research can be done to investigate the gender effect on the positive and negative emotions experienced in language learning contexts. Finally, future studies should pinpoint the relationship between EFL learners’ emotional intelligence and their disengagement in foreign language contexts.

## Author Contributions

YM the sole author confirms the final manuscript and approved its submission to Frontiers in Psychology.

## Conflict of Interest

The author declares that the research was conducted in the absence of any commercial or financial relationships that could be construed as a potential conflict of interest.

## Publisher’s Note

All claims expressed in this article are solely those of the authors and do not necessarily represent those of their affiliated organizations, or those of the publisher, the editors and the reviewers. Any product that may be evaluated in this article, or claim that may be made by its manufacturer, is not guaranteed or endorsed by the publisher.
